# Alcohol–attributed disease burden and alcohol policies in the BRICS–countries during the years 1990–2013

**DOI:** 10.7189/jogh.07.010404

**Published:** 2017-06

**Authors:** Rynaz Rabiee, Emilie Agardh, Matthew M Coates, Peter Allebeck, Anna–Karin Danielsson

**Affiliations:** 1 Karolinska Institutet, Department of Public Health Sciences, Stockholm, Sweden; 2 Institute for Health Metrics and Evaluation, University of Washington, Seattle, USA; 3 Center for Epidemiology and Community Medicine, Stockholm County Council, Stockholm, Sweden

## Abstract

**Background:**

We aimed to assess alcohol consumption and alcohol–attributed disease burden by DALYs (disability adjusted life years) in the BRICS countries (Brazil, Russia, India, China and South Africa) between 1990 and 2013, and explore to what extent these countries have implemented evidence–based alcohol policies during the same time period.

**Methods:**

A comparative risk assessment approach and literature review, within a setting of the BRICS countries. Participants were the total populations (males and females combined) of each country. Levels of alcohol consumption, age–standardized alcohol–attributable DALYs per 100 000 and alcohol policy documents were measured.

**Results:**

The alcohol–attributed disease burden mirrors level of consumption in Brazil, Russia and India, to some extent in China, but not in South Africa. Between the years 1990–2013 DALYs per 100 000 decreased in Brazil (from 2124 to 1902), China (from 1719 to 1250) and South Africa (from 2926 to 2662). An increase was observed in Russia (from 4015 to 4719) and India (from 1574 to 1722). Policies were implemented in all of the BRICS countries and the most common were tax increases, drink–driving measures and restrictions on advertisement.

**Conclusions:**

There was an overall decrease in alcohol–related DALYs in Brazil, China and South Africa, while an overall increase was observed in Russia and India. Most notably is the change in DALYs in Russia, where a distinct increase from 1990–2005 was followed by a steady decrease from 2005–2013. Even if assessment of causality cannot be done, policy changes were generally followed by changes in alcohol–attributed disease burden. This highlights the importance of more detailed research on this topic.

Alcohol consumption has been causally linked to approximately 30 diseases and injuries [1,2], and is an important risk factor to the global burden of disease [3]. Although the level of alcohol consumption varies between countries and regions, the highest per capita consumption is still found in the economically developed world [1]. However, as a result of increased economic growth, alcohol consumption is expected to increase in several low– and middle–income countries (LMIC), as has been observed in eg, Brazil and India [4].

The BRICS countries (Brazil, Russia, India, China and South Africa) are all emerging economies that have experienced increased economic growth, reduced poverty and strengthening of their health systems during the past decade [5,6]. These countries, accounting for 40% of the world’s population [5], are important and influential in global health development [7,8]. Although they have not officially signed a document declaring “we are the BRICS”, they have embraced the term and are taking steps to further develop their collaboration [8]. In 2014, the BRICS countries reiterated their commitment to prevent and control non–communicable diseases (NCDs) and to reduce the impact of risk factors on NCDs, one being harmful use of alcohol [9].

The impact of alcohol policies is well studied in several high–income countries [2,4], where eg, limited physical availability and high prices are well–established tools used to reduce alcohol consumption and related harm [2]. In contrast, such research is scarce in LMIC settings [4], and several studies focusing on the BRICS countries are calling for increased policy action to tackle alcohol consumption and subsequent harm [10,11]. This is also highlighted in the WHO Global strategy to reduce harmful use of alcohol where it is urged for action with regards to eg, drink–driving policies and countermeasures; availability of alcohol and pricing policies [12].

One increasingly used way to examine alcohol–attributed disease burden in a population and/or country, is by estimating disability adjusted life years (DALYs) [13], developed within the global burden of disease study (GBD). DALYs combine premature death or years of life lost (YLL) with years lived with disability (YLD), and thus allow for a more comprehensive assessment of disease burden. Moreover, the GBD enables comparisons across countries and over time as the disease burden is systematically and uniformly defined, and the latest methods are continuously applied for all data collected.

To date, there is no study summarizing alcohol consumption, alcohol–related disease burden and alcohol related policies in the BRICS countries. Thus, by using results from the Global Burden of Disease and Injuries and Risk Factors 2013 study, we aimed at 1) assessing overall alcohol consumption and alcohol attributed disease burden by DALYs in the BRICS countries between 1990 and 2013, and 2) identifying possible temporal linkages between evidence–based alcohol policies and alcohol consumption and alcohol–related harm.

## METHODS

### The Global Burden of Disease Study 2013

The GBD 2013 and the methods used therein have been described in detail elsewhere [13–16]. In short, GBD comprises estimates of 306 diseases and injuries, and 2337 sequelae (non–fatal health consequences of diseases and injuries) for men and women in 20 age groups, and uses DALY as measure of population health. DALYs assess years of healthy life lost by different causes and are calculated by summing YLLs (years of life lost to premature death) and YLDs (years lived with disability).

The burden of disease attributed to alcohol is estimated using a comparative risk assessment approach known as the population attributable fraction, which has been described in detail elsewhere [15,16]. Alcohol consumption has been identified as a risk factor for alcohol use disorder, self–harm and violence, transport injuries, unintentional injuries, cirrhosis, neoplasms, cardiovascular diseases, diabetes, epilepsy, pancreatitis, lower respiratory infections and tuberculosis [15].

The calculations are based on the effect of different levels of alcohol consumption on disease and injury outcome, ie, relative risks (RRs), and the prevalence of alcohol consumption at the population level. The RRs in the exposure–outcome associations are based on scientific systematic reviews and meta–analyses [16], while the distribution of alcohol consumption is based on the average all–age consumption per capita from the Food and Agriculture Organization of the United Nations (FAO) and WHO Global Information System on Alcohol and Health (GISAH) data, as well as survey data to obtain age patterns of consumption and the prevalence of drinkers, former drinkers and abstainers [16]. The RRs for some of the risk–outcome pairs for alcohol use in Russia are different from the rest of the BRICS based on evidence from a recent cohort study [16]. The contribution of alcohol to disease burden is estimated by comparing the risk of diseases or injuries under the current exposure distribution in the population (at different levels of alcohol use), to a theoretical counterfactual distribution where no one is exposed to alcohol. This population attributable fraction is then applied to the overall disease specific burden (YLLs and YLDs to later be summed, generating the DALYs).

### Evidence–based alcohol policies

Evidence–based alcohol policies, regarded as ‘best practice’ have been summarized by eg, Babor et al. (2010) [1], and include alcohol taxes, minimum legal drinking age, restriction on density of outlets, drunk driving countermeasures, and government monopoly on sales of alcohol. Information on each country’s existing alcohol policies was achieved through literature searches in the World Health Organization database, PubMed, Google Scholar and Web of Science using the following search words: 1) [(country) AND (alcohol OR alcohol consumption) AND (policy)], 2) [(BRICS) AND (alcohol OR alcohol consumption) AND (policy)].

### Analytical approach

We used the results from the Global Burden of Disease and Injuries and Risk Factors 2013 study [16] to present the age–standardized rates of DALYs attributed to alcohol, per 100 000, between 1990 and 2013 at five year intervals. Age–standardized rates adjust for total population and changes in age–specific population sizes over time, and allow comparison of alcohol–attributed health outcomes across countries. Estimates of alcohol consumption and alcohol–attributed disease burden were extracted from the GBD database provided by the Institute for Health Metrics and Evaluation (IHME) (http://www.healthdata.org/). Each policy identified through the literature searches was assessed in relation to patterns of alcohol–attributed DALYs.

## RESULTS

### Alcohol consumption and alcohol–attributable disease burden

**Figure 1:**
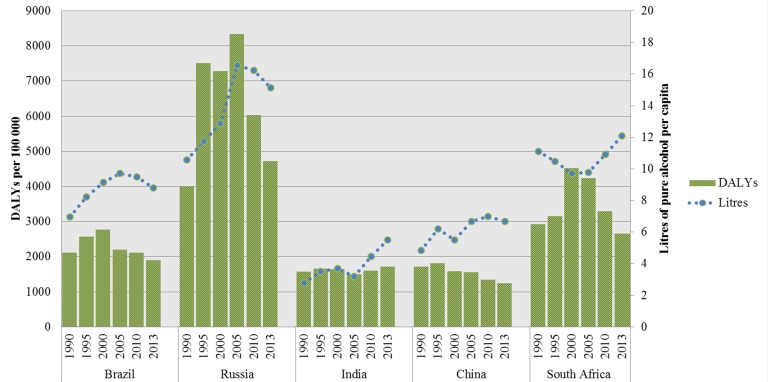
Alcohol–attributable DALYs (age–standardized per 100 000) and alcohol consumption in liters of pure alcohol in BRICS countries 1990–2013.

**Figure 1** summarizes alcohol consumption and the alcohol–attributed DALYs in the BRICS countries. The alcohol–attributable disease burden of Brazil, Russia and India mirrors their level of alcohol consumption. This is however, not the case for China and South Africa. A decreasing disease burden is observed in China and South Africa from 2000 to 2013, while, at the same time, they have rising alcohol consumption levels (ie, from 2005 an onwards for China). This is opposite to the pattern Brazil, Russia and India depict.

### Alcohol policies

The implemented evidence–based alcohol policies in each country are summarized in **Table 1**. All BRICS countries have implemented excise tax on alcohol and drink–driving countermeasures. All countries also have regulations on exposure (advertisement, product placement etc.). India is the only country out of the five BRICS where restrictions on density of outlets are implemented and all countries barring Brazil have time and day restrictions on sales. China is the only country that has not implemented a legal minimum drinking age. None of the countries has a government monopoly on the sale of alcohol.

**Table 1:** The identification of implemented evidence–based polices in the BRICS countries (year of implementation in brackets)*

**Policy**	Brazil	Russia	India	China	South Africa
Alcohol taxes	Yes (2004) [17]	Yes (2000), (2008) [18]	Yes (2004) [17]	Yes (2002) [19]	Yes (2003) [20]
Minimum legal drinking age	Yes (1999)	Yes (2001) [18]	Subnational (2004) [17]	No	Yes (2004) [17]
Time and day restrictions on sales	No	Yes, time (2005), (2006) [18]	Yes (2004) [17]	Yes (2004) [17]	Yes, time (2003) [20]
Restrictions on density of outlets	No	No	Yes (2004) [17]	No	No
Drink–driving countermeasures	Yes (2008) [11]	Yes (1999) [21]	Yes (1999] [21], (2004) [17]	Yes (2007) [19]	Yes (1999) [21]
Legal restriction on exposure	Yes, ads (1996) [21]	Yes, ads (1993), (1995), (2001) [18]	Yes (2004) [17]	Yes, ads (2010) [22]	Yes, ads (2004) [20]
Government monopoly	No	No (removed 1992) [18]	No	No	No

*Where year of implementation is not available; year when policy is noted to be in place is used instead.

### Alcohol policies in relation alcohol–attributable disease burden

**Figure 2:**
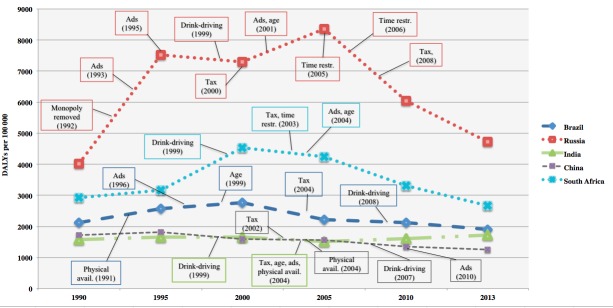
Change in alcohol attributable DALYs over time and implemented policies (year).

In 1992, Russia removed the state monopoly and in 1995 there was an increase in alcohol–attributable DALYs (**Figure 2**). Bans on advertisement and alcohol taxes were implemented (1993–2000), and from 1995–2000 the DALYs were rather constant. Between 2000 and 2005 there was a slight increase in DALYs (from 7288 to 8349 DALYs per 100 000). Russia implemented further alcohol policies on requirements on licensing and restriction on sales (2005–2006), after the legal drinking age was set in 2001, and then increased taxes (in 2008). Between 2005 and 2013 there was a substantial decrease in alcohol attributable DALYs (8349 to 4719 DALYs per 100 000).

In 1991, Brazil increased the flexibility of regulating sales and at this time DALYs were modestly increasing (2124 to 2771 DALYs per 100 000 between the years 1990–2000) (**Figure 2**). In the year 1997, the blood–alcohol concentration limit while driving was lowered, and from the year 1998 to 2008 the limit was set to zero (“Prohibition”), to later in 2008 be re–established and set to 0.02 g/L (grams per liter).

In South Africa, policies on taxes and restriction on sales were implemented in 2003 and advertisement was restricted in 2004. In 2008, a liquor bill was implemented, which regulated the physical availability of alcohol and in 2009 messages on container labels came into place. The DALYs peaked in the year 2000 in South Africa (4527 DALYs per 100 000), and an overall decrease was observed over the studied time period (2926 to 2662 DALYs per 100 000 between the years 1990–2013).

In China, the taxes on alcohol increased in 2002, and in 2004 restrictions on sales and licensing were in place. In 2007, measures on drunk driving were implemented and in 2010 restrictions in advertisement were in place. As in South Africa, an overall decrease in alcohol–attributable DALYs was observed in China (1719 to 1250 DALYs per 100 000 between the years 1990–2013).

In India, alcohol policies were observed to be in place for the years 1999 (the blood alcohol concentration while driving was set as 0.10 g/L) and 2004 (taxes, minimum legal age and limitations on physical availability and exposure). The DALYs increased over time in India (from 1574 to 1722 DALYs per 100 000 from 1990–2013) with a modest decline between the years 2000 and 2005 (from 1647 to 1512 DALYs per 100 000).

### Discussion

Our study showed an overall decrease of age–standardized disability adjusted life years (DALYs) in Brazil, China and South Africa, while an overall increase was observed in Russia and India when comparing 1990 to 2013. Most notably is the change in DALYs in the case of Russia, where a distinct increase between 1990 and 2005 was followed by a steady decrease from 2005 to the year 2013. For Brazil, India and Russia, levels of alcohol consumption were in line with levels of alcohol–attributed disease burden, while in China these levels separated from 2005 and onwards, and in South Africa the levels are totally the opposite of each other during the entire time period. Alcohol policies are implemented in all the BRICS countries, although types of policies differ. Furthermore, our results show that policy changes are generally followed by changes in alcohol–attributed disease burden.

Although Russia is part of the BRICS, their economic development is quite different [5] from the rest of the BRICS countries, as is their alcohol history; from the fall of the Soviet Union with the removal of state monopoly in 1992 [23], the recovering economic development in Russia in 2000, when alcohol consumption and alcohol–attributed disease burden increased, to the alcohol policies implemented in 2006 followed by a drop in alcohol–attributable DALYs in 2010. The major causes of deaths in Russia in the late 90s were cardiovascular diseases, cancer and violent causes [24]; ie, conditions where alcohol consumption is a known risk factor. Through the transition period in the beginning of 1990, alcohol related deaths increased the most [25]. The distinguished change of alcohol–related deaths in Russia has been observed earlier [24] and the history of Russia in the early 90s is worth highlighting when comparing the results to the other BRICS countries. It has been suggested that the drop seen in DALYs (year 2005 and onwards) was a result of Gorbachev’s reform in 1985 (ie, raising prices and restricting sales). Some additional positive changes in alcohol consumption seem to be a result of the 2006 policy changes in Russia (eg, restricting sale locations and regulations on licensing for producers and distributors) [18,23,26].

India was the one country besides Russia that experienced an overall increase in alcohol–attributable DALYs between 1990 and 2013. India is a large and diverse country with regards to both age and gender compositions across the country and with vast differences in drinking patterns as well as adopted policies in different areas [27,28]. Of the few studies conducted on this topic in India, prevalence reports on alcohol consumption differ largely [29]. Consequently, as suggested by Girish et al. [28], different strategies to prevent and control alcohol are needed in different areas. At the national level, the policies in India (eg, taxes, minimum legal drinking age and drunk driving measures) were found to be in place in 1999 and 2004, slightly before the observed increase in disease burden, which might partially be explained by the increase in alcohol consumption from 2005 and onwards.

In the case of Brazil, the alcohol–attributable burden of disease decreased over time. Drinking and driving limitation and age restrictions are among the implemented alcohol policies in Brazil. A previous study showed that the 2008 policy implementation in Brazil (drunk driving) had a significant impact on reduction of traffic injuries and fatalities [30]. Other studies conducted in Brazil have emphasized comprehensive and effective alcohol and drug policy [11] since so far policies have been fragmented and poorly enforced [31]. As shown by our results, the alcohol consumption and the attributable disease burden mirror each other and the implemented policies fit the trend of the decreasing disease burden.

Although the alcohol–attributable DALYs in China have decreased over the decades, recent studies show an alarming increase in alcohol consumption and related harms [10,32], as well as an expansion of the alcohol production [10]. The lack of comprehensive alcohol policies [19,32] has been emphasized, as has the limited research on alcohol policy in China for the past decades [19]. Since DALYs have decreased in China despite the fact that the country has rather few implemented policies, it seems relevant to raise the question of a possible lagging effect of the implemented policies on the alcohol–attributable burden of disease.

Like Brazil and China, South Africa has experienced a decrease in alcohol–attributable DALYs. In South Africa there are similar diversities within the country as in India and drinking patterns differ largely in different areas or states [33,34]. An increase in alcohol consumption especially among youth, and an increase in traffic accidents and violence have been observed in the past two decades [34]. In South Africa, the development in alcohol consumption is almost the exact opposite to the DALY development. This peculiar trend can be explained by several phenomena. First, the consumption estimates plotted are total per capita, while the burden estimates are age–standardized. Second, in the comparative risk assessment approach, age–sex–specific attributable fractions are applied to the outcome–specific total burden, so the total burden trends can affect trends in attributable burden if the attributable fraction component changes relatively less than the total burden. In South Africa, the total age–standardized rate of DALYs rose between 1990 and 2000 and fell between 2000 and 2013 for many causes in which alcohol is a risk factor (eg, transport injuries, cirrhosis, tuberculosis, stroke, hypertension, and self–harm and interpersonal violence). Hence, different patterns emerge when results are stratified by age, sex, outcome (eg, liver cancer, ischemic stroke, etc.) and metric (YLL/YLD).

### Strengths and limitations

The BRICS countries are some of the world’s largest countries, both with regard to population size [6] and land coverage [35], and in some cases different legal and policy regulations in different states within the countries [10,11,27]. Our study provides a first step of a cross–country comparison within the field of alcohol research in these countries. To compare the results from this study with research conducted on alcohol consumption and alcohol policy in high–income countries, where established systems for surveillance and monitoring exists, might be quite simplifying and deceptive because of the different contexts. With that in consideration, this study is in line with previous studies conducted in LMIC, emphasizing the need for empirical research on levels of alcohol consumption and effects of, as well as adherence to, implemented policies [10,11,20,33,34].

We recognize that many factors contribute to and explain the alcohol related disease burden, and an assessment of causality between implemented policies, alcohol consumption and attributable burden obviously cannot be done. For one thing, we do not know to what extent the alcohol policies were adequately enforced. A recent study by Ferreira–Borges and colleagues [36] focusing on alcohol policies in 46 African countries highlighted the need for increased training and capacity building among government leaders and decision makers in the development and implementation of alcohol control policies. There is also the possibility of differences in time lags for the different policies with regards to effects on consumption and harm. For example, the implementation of drink–driving countermeasures and increased pricing may have more immediate effects on alcohol–related harm than restrictions on advertisements.

Furthermore, there are limitations regarding the quality and validity of data. Per capita consumption tends to be underestimated and for this purpose a correction factor is used in the GBD calculation to account for unrecorded consumption [16]. Also, alcohol–attributed deaths tend to be underreported in registers due to difficulties in making accurate diagnoses. Coding practices also differ across countries and although the GBD study uses a general approach to assess causes of deaths from all countries, little is known about to what extent differences in coding may affect the estimates.

Our information on policies derives from published literature only and it is possible that we lack information on some alcohol policy changes that may have taken place in the BRICS countries during this time period. The alcohol policies included in this study are only those that have been documented to be effective [2]. However, most research building the evidence–based compilation [1] is based on studies in high–income countries (HIC). There may be context–specific factors in LMIC demanding different alcohol policies or interventions, as opposed to what has been shown in HIC. The lack of evaluations of current policies in the BRICS highlights the importance of further research.

The comparison carried out in this study is, to our knowledge, the first to be made. A key strength with the GBD methodology is that disease burden due to alcohol is systematically and uniformly defined and thus estimates can be compared across countries and over time. The results from this study illustrate a pattern in the development of alcohol–attributable disease burden and alcohol policy over time for the BRICS countries. Country specific studies on overall disease–burden have been conducted in Germany [37], the Kingdom of Saudi Arabia [38], Spain [39], and the United Kingdom [40], and specifically on alcohol–related disease burden in the Nordic countries [41] where GBD data for each country has been utilized to assess cross–country similarities and differences in relation to policy (eg, alcohol). Similar studies are yet to be conducted in LMIC. As regional differences have been highlighted in our study, the on–going subnational burden of disease analyses conducted in Brazil, China, India, and South Africa are highly relevant, where regional differences in alcohol–attributable disease burden are more likely to be captured, in turn shedding more light on this topic.

## CONCLUSIONS

The alcohol–attributable DALYs changed between the years 1990–2013, with an overall increase in Russia and India and a decrease in Brazil, China and South Africa. This reflected the alcohol consumption development quite well for Brazil, Russia, and India, but only partly for China. However, the alcohol consumption in South Africa was roughly the opposite of the disease burden development for several reasons. Types of implemented alcohol policies varied between the countries, however all countries had policies on alcohol taxes, drink driving and advertisement.

Our study provides a first step of a cross–country comparison within the field of alcohol research in these countries, highlighting the need for further empirical research on levels of alcohol consumption and subsequent harm, and effects of, as well as adherence to, implemented policies.
